# Association of Plaque Characteristics With New Ischemic Lesions After Carotid Artery Stenting

**DOI:** 10.1111/cns.70312

**Published:** 2025-03-03

**Authors:** Senhao Zhang, Mengmeng Feng, Fan Yu, Xin Meng, Yue Zhang, Bixiao Cui, Tao Wang, Weizhao Lu, Hongwei Yang, Shaozhen Yan, Jie Lu

**Affiliations:** ^1^ Department of Radiology and Nuclear Medicine Xuanwu Hospital, Capital Medical University Beijing China; ^2^ Beijing Key Laboratory of Magnetic Resonance Imaging and Brain Informatics Beijing China

**Keywords:** carotid stenosis, new ischemic lesions, PET/MR, vulnerable plaque

## Abstract

**Background:**

Carotid artery stenting (CAS) is a common treatment for carotid artery stenosis, but it can lead to new ischemic brain lesions on diffusion‐weighted images (DWI) during the perioperative period. Identifying these lesions early is crucial to preventing recurrent ischemic strokes.

**Methods:**

This retrospective study included 47 patients who underwent CAS. Preoperative carotid PET/MR examinations and postoperative brain MRI were performed. Clinicians identified the responsible carotid artery based on symptoms and records. Vessel morphology, plaque characteristics, and inflammatory uptake were analyzed. The standardized uptake value and tissue‐to‐background ratio quantified ^18^F‐fluorodeoxyglucose(^18^F‐FDG) uptake. The symptomatic carotid atheroma inflammation lumen‐stenosis(SCAIL) score assessed stenosis severity and inflammation. The primary outcome was the presence of new ischemic lesions on DWI.

**Results:**

Among the 47 patients (mean age, 65 ± 7 years; 44 males), 30 (63.8%) exhibited new ischemic lesions. These patients had a higher prevalence of AHA type VI plaques (50.0% vs. 17.6%, *p* = 0.028), higher PET uptake (43.3% vs. 11.8%, *p* = 0.026), and higher SCAIL scores (63.3% vs. 23.5%, *p* = 0.009). The most common distribution pattern of new ischemic lesions was located in the mixed (in and beyond of the treated artery) territory (36.2%). Of the 30 participants with new ischemic lesions, 15 (50%) had lesions located in both peripheral brain areas and deep brain areas. In the adjusted model, high PET uptake and SCAIL scores were independently associated with new ischemic lesions (aOR = 7.26, 95% CI: 1.22, 73.59; *p* = 0.049 and aOR = 7.06 [95% CI: 1.50, 44.18]; *p* = 0.020).

**Conclusion:**

Carotid PET/MR‐related indicators can effectively predict the risk of new ischemic lesions on DWI during the perioperative period after carotid artery stenting, providing important references for early identification of high‐risk patients for recurrent ischemic stroke. Further large‐scale randomized controlled studies are necessary to validate the clinical application value of these indicators.

## Graphical abstract

Carotid PET/MR‐related indicators (high PET uptake, high SCAIL scores, AHA VI type plaques) can effectively predict the risk of new ischemic lesions after carotid artery stenting, aiding in the early identification of high‐risk patients for recurrent ischemic stroke.

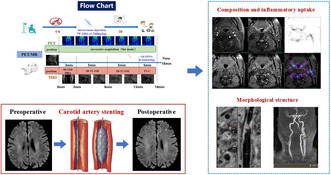

**Abbreviations**
CAS: Carotid Artery Stenting
DWI: diffusion‐weighted Images
FDG: fluorodeoxyglucose
IPH: intraplaque hemorrhage
MRI: magnetic resonance imaging
PET: positron emission tomography
SCAIL: carotid atheroma inflammation lumen‐stenosis
TBR: target‐to‐background ratio
TIA: transient ischemic attacks

## 1 Introduction

Atherosclerotic carotid artery stenosis is a significant cause of ischemic stroke or transient ischemic attacks (TIA) compared to other stroke subtypes [1]. Carotid artery stenting (CAS) has emerged as a crucial alternative treatment for carotid artery stenosis. An international study on carotid artery stenting indicated that the incidence of periprocedural ipsilateral ischemic lesions is higher in CAS than in carotid endarterectomy (CEA) [2, 3, 4]. Endovascular procedures, including diagnostic angiography and CAS, often result in clinically silent new ischemic brain lesions, which occur more frequently than expected [5, 6, 7]. Although these ischemic lesions are frequently asymptomatic, previous studies suggest they may contribute to subtle cognitive changes or other long‐term consequences that warrant further investigation and may serve as surrogate markers for future stroke recurrence [8, 9]. Identifying high‐risk patients for new ischemic lesions post‐CAS aids in devising personalized treatment strategies, and these lesions can be monitored through magnetic resonance imaging (MRI).

Traditionally, the degree of luminal stenosis measured by ultrasound has been the primary morphological marker for selecting vulnerable plaques for treatment. However, studies have shown that even among patients with similar degrees of luminal stenosis, there are differences in absolute risk reduction, underscoring the importance of factors beyond luminal stenosis in risk assessment [10, 11]. The advent of high‐resolution MRI has enabled the identification of various compositional biomarkers, such as intraplaque hemorrhage (IPH), lipid‐rich necrotic core, and irregular plaque surface, to characterize the vulnerability of plaques [12]. Previous carotid MRI studies [13, 14, 15] in symptomatic carotid stenosis patients have demonstrated that type VI lesions in the AHA classification or high‐risk plaque features, particularly IPH, are associated with an increased risk of recurrent ischemic stroke or TIA and distal embolization post‐CAS [16]. However, even with the aid of machine learning, diagnostic tests based on single imaging biomarkers have shown only moderate validity, highlighting the need for more advanced assessments.

Recent research has established inflammation as a critical factor in the instability of atherosclerotic plaques, shifting imaging research toward inflammation assessment [17]. Currently, ^18^F‐FDG is the most widely used molecular imaging agent for atherosclerosis, capable of non‐invasively imaging inflammation‐related metabolic activity in carotid plaques. Studies have shown that ^18^F‐FDG uptake in carotid plaques correlates with other features of plaque instability, such as blood inflammatory markers, IPH, and lipid‐rich core [18]. Prior clinical studies have also indicated that ^18^F‐FDG uptake in symptomatic carotid plaques is associated with early recurrent stroke, independent of luminal stenosis [19]. While PET is valuable in assessing plaque inflammation, its standalone or combined CT imaging capability for compositional evaluation is limited. Integrated PET/MR technology allows simultaneous measurement of PET activity and plaque composition. The incremental value of PET over MRI in evaluating carotid plaques remains unclear.

In this study, we aim to utilize integrated PET/MR to simultaneously assess the morphological and metabolic characteristics of atherosclerotic carotid plaques and explore its value in predicting early new ischemic brain lesions post‐CAS. Through this study, we hope to provide new evidence for the application of advanced imaging biomarkers in risk stratification, thereby improving the clinical management of patients undergoing CAS and reducing the incidence of recurrent ischemic strokes.

## 2 Materials and Methods

### 2.1 Patient Selection and Clinical Assessment

This study was approved by the Ethics Committee of our hospital (Approval Number: KS2022023‐1). Written informed consent was obtained from all participants or their legal guardians. From September 2020 to May 2023, patients with carotid artery stenosis admitted to the Department of Neurosurgery at our hospital were consecutively enrolled. All patients received endovascular treatment upon admission, and those who underwent carotid artery stenting (CAS) were included in this study. The inclusion criteria were as follows: (1) Eligibility for CAS versus carotid endarterectomy (CEA) was determined based on the North American Symptomatic Carotid Endarterectomy Trial (NASCET) criteria (i.e., asymptomatic carotid stenosis > 70% or symptomatic carotid stenosis > 50%) [20]. In addition, factors such as patient preference, medical comorbidities, lesion anatomy, and the relative risk of surgical versus endovascular intervention were considered. Patients who were deemed suitable candidates and opted for CAS were included in this study; (2) participants who failed maximal medical therapy, including dual antiplatelet therapy and intensive risk factor management; (3) those who underwent integrated PET/MR of the carotid artery within 1–3 days before CAS, with brain MRI on the same day as the PET/MR, and another brain MRI within 3 days post‐CAS. The exclusion criteria were: (1) incomplete clinical data; (2) participants with non‐atherosclerotic stenosis, such as dissection, arteritis, and Moyamoya disease; (3) participants with intracranial aneurysms in the target artery; (4) uncooperative participants or those with contraindications to MRI; (5) poor quality of PET/MR or MR images (score < 3). After screening, data from 47 eligible patients were collected (screening procedure detailed in Supporting Information).

Clinical and laboratory data were collected from patients upon admission. Baseline demographic data included age, sex, and BMI. Stroke risk factors included hypertension, hyperglycemia, hypercholesterolemia, hyperlipidemia, history of coronary heart disease, and smoking status. Laboratory data obtained within 24 h of admission included high‐sensitivity C‐reactive protein (CRP) and homocysteine. Additionally, essential baseline data, such as whether the patient was symptomatic and their previous medication use, were collected.

### 2.2 MRI Protocols

All enrolled participants underwent carotid artery integrated PET/MR and brain MR examinations 1–3 days before and after endovascular treatment. All patients were asked to fast for at least 6 h before ^18^F‐FDG PET imaging. Imaging was performed only if fasting glucose was lower than 7.7 mmol/L before tracer injection. ^18^F‐FDG was injected intravenously at a dose of 3–4 MBq/kg. The acquisition started 90–120 min after tracer injection using an integrated PET/MRI system (uPMR790, United Imaging Healthcare, Shanghai, China). MR vessel wall imaging was performed with an 8‐channel carotid coil. The PET/MR image acquisition range was centered on the carotid bifurcation, with an 18 cm coverage.

High‐resolution carotid vessel wall MRI sequences included 3D TOF‐MRA, 3D T1, 3D T2, and post‐contrast (gadolinium‐DTPA, 0.1 mmol/kg, 2.5 mL/s) enhanced 3D T1 (T1C+). Specific parameters were as follows: (1) three‐dimensional time of flight (3D‐TOF), TR/TE(ms) = 17.5/4, FOV(mm) = 180*180, resolution (mm) = 0.75*0.60*1.5, slice number = 323;(2)3D T1 FSE, TR/TE(ms) = 800/14.52, FOV(mm) = 180 mm*180 mm, resolution(mm) = 0.60*0.60*0.60, slice number = 390;(3)3D T2 FSE, TR/TE(ms) = 2000/198.44, FOV(mm) = 180*180, resolution (mm) = 0.60*0.60*0.60, slice number = 390, (4)3D T1 FSE + C, TR/TE(ms) = 800/14.52, FOV(mm) = 180*180, resolution (mm) = 0.60*0.60*0.60, slice number = 390. PET images were reconstructed using an OSEM algorithm (matrix size = 256 × 256, thickness = 1.4 mm) with time‐of‐flight information following data corrections for attenuation, scatter, and random coincidences. Brain MRI Scanning was performed using GE Discovery 750 W. Routine brain MRI sequences included axial T1WI, T2WI, T2‐FLAIR, and DWI, covering the range from the cranial vertex to the foramen magnum. DWI sequence parameters for the GE Discovery 750 W scanner: TR/TE = 3672/77.9 ms, FOV(mm) = 240 × 240 mm, Acquisition Matrix = 128 × 128, Slice thickness(mm) = 5, Flip angle(°) = 90, with *b* = 0, 1000s/mm^2^.

### 2.3 Analysis of FDG PET/MR Images

PET/MR image analysis was conducted using a dedicated plaque analysis workstation (United Imaging Healthcare, Shanghai, China). The quality of the image was rated on a 4‐point scale (1 = poor and 4 = excellent), determined by the clarity of the arterial wall, luminal margin, and plaque components [21]. Patients with an image rating < 3 were excluded. Three experienced neuroradiologists (SH Z, MM F, and FY; each with over 10 years of experience) analyzed the MR images independently and were blinded to all other information. Two (SH Z and MM F) independently reviewed the images, with discrepancies resolved by a third reviewer (FY). Plaque composition and surface status were classified based on AHA lesion types [22] (Table S1). The focus was on determining if a plaque was classified as AHA‐LT VI and documenting these findings. For quantitative analysis, the degree of stenosis (NASCET), plaque area, and remodeling index were calculated at the most stenotic slice. By delineating the circular regions of interest for each slice, the maximum standardized uptake value (SUVmax) of the plaque was calculated; SUVmax ≥ 2.85 was considered indicative of high PET uptake [23]. The maximum target‐to‐background ratio (TBRmax) was calculated as the ratio of SUVmax to the venous blood pool SUVmean [24]. Inflammation levels were categorized based on TBR values: mild (TBR = 1.25–2), moderate (TBR = 2–3.2), and severe (TBR > 3.2) [25]. The symptomatic carotid atheroma inflammation lumen‐stenosis(SCAIL) score was calculated for each patient, utilizing a 2‐item scale that includes lumen stenosis and plaque inflammation (Table S2). Patients were categorized into low SCAIL score (< 3) and high SCAIL score (> 3) groups [26].

### 2.4 CAS Procedure Protocols

All CAS procedures were executed by the same surgical team under local anesthesia via the femoral artery approach. Patients were pre‐treated with daily doses of clopidogrel and aspirin for antiplatelet therapy. Intraoperatively, intravenous heparin (100 U/kg) was administered for anticoagulation. Digital subtraction angiography (DSA) was used prior to stent placement to assess blood flow and stenosis. A 6‐8F catheter was navigated to the ipsilateral common carotid artery proximal to the stenosis. After stent deployment, residual stenosis was confirmed to be less than 30%.

### 2.5 Evaluation of New Ischemic Lesions

Two radiologists (SH Z and MM F), blinded to clinical data and endovascular treatment details, independently reviewed all pre‐ and post‐intervention DWI and apparent diffusion coefficient (ADC) maps. Acute periprocedural ischemic lesions were defined as new diffusion‐restricted lesions (hyperintensity on DWI and hypointensity on ADC) appearing post‐treatment but absent pre‐treatment [5, 27]. New ischemic lesions were categorized into symptomatic ischemic strokes and asymptomatic new lesions. The frequency of any new ischemic lesions post‐carotid stenting was recorded. We further analyzed the distribution, location, and number of new lesions post‐carotid stenting. The distribution of new lesions was classified into ischemic lesions within the treatment vessel territory and those outside the treatment artery territory [27]. Lesion locations were categorized into peripheral brain regions supplied by the main branches of the carotid artery (including cortex and subcortical white matter) and deep brain regions supplied by perforating arteries [28]. The total number of new lesions per patient was also recorded. Ischemic lesions were considered independent if there was no continuity between lesions on the same slice and adjacent slices [29].

### 2.6 Statistical Analysis

Descriptive statistics are expressed as frequencies and percentages for categorical variables, while continuous variables are represented by mean (SD) or median (IQR), depending on suitability. The intraclass correlation coefficient (ICC) was calculated to assess the reproducibility of SUVmax, TBRmax, and the count of new ischemic lesions between raters. An ICC greater than 0.75 was considered to indicate good to excellent reliability. We first assessed the normality of all continuous variables using the Shapiro–Wilk test. For variables that followed a normal (Gaussian) distribution, results are reported as mean ± standard deviation, and differences were compared using the Student's t‐test. For variables that did not pass the normality test, we report data as medians (interquartile range [IQR]) and employed nonparametric tests (e.g., Mann–Whitney U test, Kruskal‐Wallis test) for comparisons. Categorical variables were expressed as numbers (percentages), and a P‐value < 0.05 was considered statistically significant.

Bivariate and multivariate logistic regression analyses were conducted to determine how PET/MR evaluation metrics and other characteristics relate to the perioperative new ischemic lesions. Three multivariable logistic regression models were constructed, adjusting for clinically relevant covariates and potential confounders [30, 31]. In the first predictive scheme, we focused on two indicators—high PET uptake and high SCAIL score—and assessed their associations under each model: Model 1 represents the unadjusted (single‐factor) analysis. Model 2 adjusts for demographic and cardiovascular risk factors (e.g., sex, age, hypertension, diabetes, hypercholesterolemia). Model 3 includes the covariates in Model 2 and imaging variables of plaque and vascular walls: plaque burden, positive remodeling, and type VI plaque. A *p* < 0.05 was considered statistically significant. Statistical analyses were performed using IBM SPSS (version 26.0; IBM Corporation).

## 3 Result

### 3.1 Comparison of Patient Characteristics Between Groups

As shown in Figure S1, 47 CAS‐treated patients met the inclusion criteria after screening. The mean age of the subjects was 65 ± 7 years, with 44 males (93.6%) and 28 out of 47 (59.6%) having symptomatic plaques. Postoperatively, 30 patients (63.8%) developed new ischemic lesions. The inter‐observer and intra‐observer consistency for radiological feature measurements was high (inter‐group correlation coefficient, 0.95; intra‐group correlation coefficient, 0.88).

**FIGURE 1:**
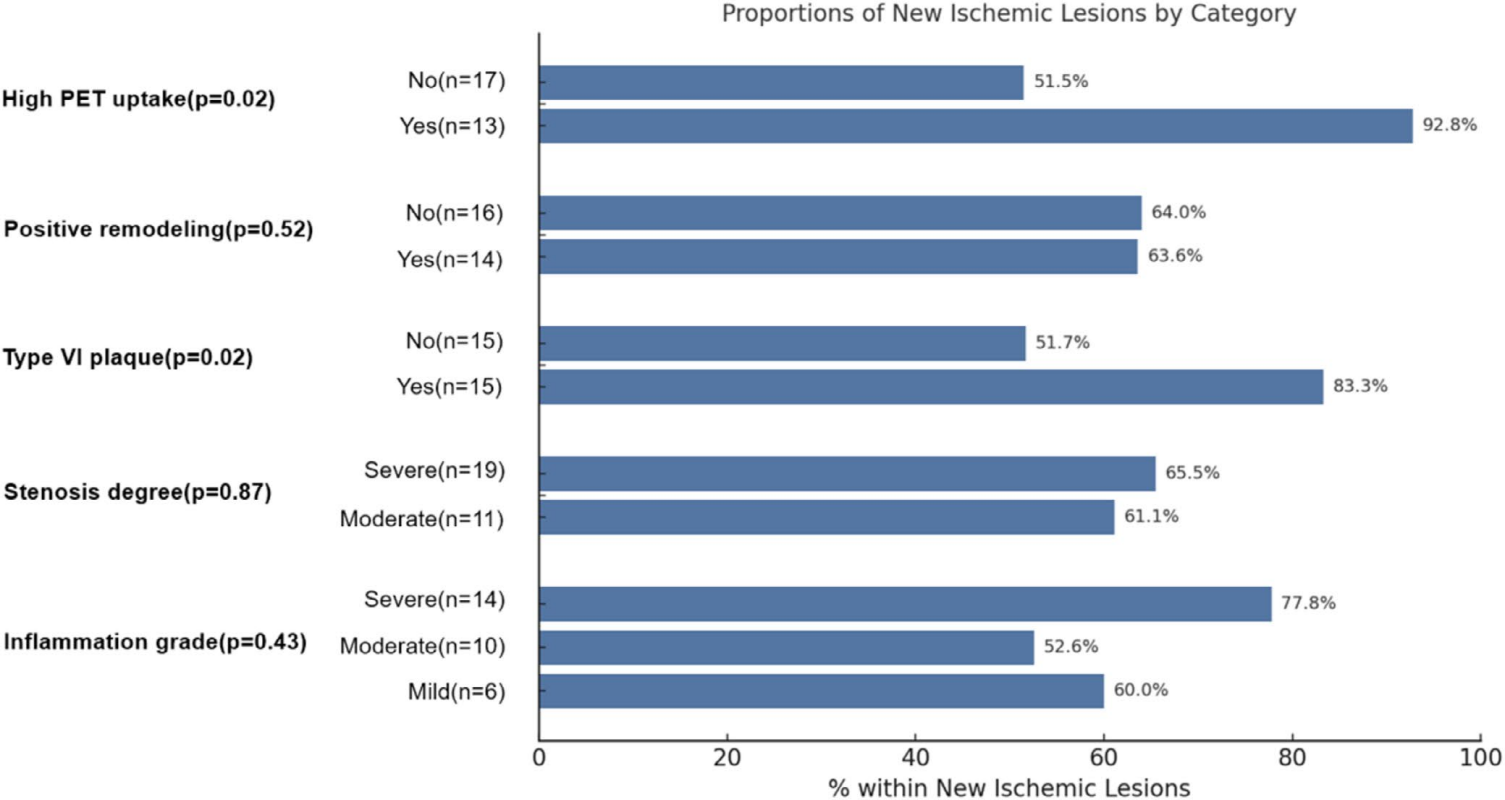
Bar graph shows data correlation with new ischemic lesions with percentage of participants by PET high uptake and markers of high risk.

Comparing the groups with and without new ischemic lesions (Table 1), there were no significant differences in cardiovascular risk factors, clinical and treatment profiles, or laboratory indicators (*p* > 0.05). In terms of angiographic profiles, there were no significant differences in lumen diameter and lumen area between the two groups. Similarly, there was no significant difference in plaque burden (0.95 [0.90, 0.97] vs. 0.93 [0.87, 0.96]), positive remodeling (46.7% vs. 47.1%), and severity of stenosis (63.3% vs. 58.8%). The individual features of vulnerable plaques (lipid‐rich necrotic core, hemorrhage, calcification, and ulceration) did not show significant differences between the groups. However, the proportion of AHA‐VI plaques was significantly higher in the new ischemic lesions group compared to the no new ischemic lesions group (50.0% vs. 17.6%, *p* = 0.028). To further investigate which specific features of these AHA VI plaques might drive this association, we analyzed the prevalence of IPH, ulceration, and thrombosis within this subgroup. Our data indicate that IPH was the most common feature (13/18, 72.2%) and appears to contribute most to postoperative new ischemic lesions (40% vs. 17.6%, *p* = 0.114). Among PET‐related metrics, the new ischemic lesions group had a higher proportion of high PET uptake (43.3% vs. 11.8%, *p* = 0.026) and high SCAIL score (63.3% vs. 23.5%, *p* = 0.009) compared to the no new ischemic lesions group, though the inflammation grade did not show a significant difference(Figure 1).

**TABLE 1:** Patient demographics and baseline characteristics between groups.

Characteristic	New ischemic lesions		*p* ^b^
	No, *N* = 17^a^	Yes, *N* = 30^b^	
Gender (Male%)	16 (94.1)	28 (93.3)	0.999
Age (years)	66 ± 8	65 ± 6	0.612
BMI	25.1 ± 3.0	25.2 ± 3.4	0.886
Risk factors			
Smoking status (%)			0.862
Never	7 (41.2%)	13 (43.3%)	
Former	5 (29.4%)	7 (23.3%)	
Current	5 (29.4)	10 (33.3)	
Hypertension (%)	9 (52.9)	24 (80.0)	0.051
Diabetes (%)	7 (41.2)	14 (46.7)	0.716
Hypercholesterolemia (%)	1 (5.9)	2 (6.7)	0.999
Hyperlipidemia (%)	7 (41.2)	17 (56.7)	0.307
Coronary artery disease (%)	7 (41.2)	6 (20.0)	0.176
Clinical and treatment profiles			
Symptomatic (%)	9 (52.9)	19 (63.3)	0.485
Medication history (%)			0.437
Antiplatelet + statin therapy	11 (64.7)	20 (66.7)	
Statin therapy	5 (29.4)	5 (16.7)	
No treatment	1 (5.9)	5 (16.7)	
Laboratory profiles			
High‐sensitivity C‐reactive protein (mg/L)	1.2 (0.6, 3.1)	1.0 (0.5, 3.6)	0.806
Homocysteine (umol/L)	14 (11, 15)	15 (12, 19)	0.224
Angiography profiles			
Lumen diameter (mm)	1.65 (1.17, 2.02)	1.42 (0.82, 1.83)	0.335
Lumen area (mm^2^)	2.9 (2.2, 6.0)	2.5 (1.6, 5.6)	0.339
Plaque burden	0.93 (0.87, 0.96)	0.95 (0.90, 0.97)	0.430
Positive remodeling (%)	8 (47.1)	14 (46.7)	0.979
Stenosis degree (Severe%)	10 (58.8)	19 (63.3)	0.760
Plaque characteristics			
Lipid‐rich necrotic core (%)	10 (58.8)	22 (73.3)	0.305
Hemorrhage (%)	3 (17.6)	12 (40.0)	0.114
Calcification (%)	9 (52.9)	15 (50.0)	0.846
Ulceration (%)	1 (5.9)	7 (23.3)	0.228
AHA‐VI plaque (%)	3 (17.6)	15 (50.0)	0.028^**c**^
High PET uptake (%)	2 (11.8)	13 (43.3)	0.026^**c**^
Inflammation grade (%)			0.307
Mild	4 (23.5)	6 (20.0)	
Moderate	9 (52.9)	10 (33.3)	
Severe	4 (23.5)	14 (46.7)	
High SCAIL score (%)	4 (23.5)	19 (63.3)	0.009^**c**^

*Note:* Values are given as Mean ± standard deviation or median [IQR] for continuous data and as frequency in percentage (*n*) for categorical data.

Abbreviation: SCAIL, symptomatic carotid atheroma inflammation lumen–stenosis.

^a^ *n* (%); Mean ± SD; Median (IQR).

^b^ Fisher's exact test; Welch Two Sample t‐test; Wilcoxon rank sum test; Pearson's Chi‐squared test; Wilcoxon rank sum exact test.

^c^ Statistically significant (*p* < 0.05).

### 3.2 New Ischemic Lesions After CAS Treatment

We analyzed 30 new ischemic lesions to determine the pathophysiological mechanisms of perioperative new ischemic lesions. Overall, 65% of participants developed new ischemic lesions, with 28 patients (59.6%) having asymptomatic new ischemic lesions and 2 patients (4.3%) experiencing TIA or ischemic stroke post‐treatment. The first patient exhibited a standardized wall index (plaque burden) of 0.80, indicative of positive remodeling, along with a lipid‐rich plaque featuring partial hemorrhage. The second patient presented with a standardized wall index (plaque burden) of 0.84, also demonstrating positive remodeling, and plaques characterized by both hemorrhage and ulceration. The most common pattern in the cohort was new ischemic lesions in both peripheral brain areas and deep brain areas (15 participant, 50%), followed by lesions in peripheral brain areas only (9 participant, 30%), and new lesions in Deep brain areas only (6 participant, 20%). Regarding the distribution of new lesions, 25.5% (12 participant) had lesions only in the territory of the treated artery, while 2% had lesions entirely beyond the treated artery's territory. A larger proportion, 36.2% (17 participant), had mixed territory ischemic lesions (Table 2). A total of 98 new ischemic lesions were identified among the 30 participants, with a median of 2 lesions per participant (IQR 2 to 4.75). Of these, 61 lesions (62.2%) were located in the territory of the treated artery (median of 2 lesions per participant, IQR 1 to 2.75), and 37 lesions (37.8%) were beyond the territory of the treated artery (median of 1 lesion per participant, IQR 0 to 2).

**TABLE 2:** New Ischemic lesions detectable at diffusion‐weighted imaging after carotid artery stenting treatment.

Characteristic	No. of participants
New ischemic brain lesions	30 (63.8)
Symptomatic ischemic stroke	2 (4.3)
Asymptomatic new lesions	28 (59.6)
Distribution of new lesions	
In the territory of the treated artery only	12 (25.5)
Beyond the territory of the treated artery only	2 (4.3)
In the mixed territory	17 (36.2)
Location of new lesions^a^	
Peripheral brain areas only	9 (30.0)
Deep brain areas only	6 (20.0)
Peripheral and deep brain areas	15 (50.0)
Total new lesion count^b^	98
In the territory of the treated artery	61 (62.2)
Beyond the territory of the treated artery	37 (37.8)
No. of new lesions per participant	2 (2,4.75)
No. of new lesions in the territory of the treated artery per participant	2 (1,2.75)
No. of new lesions beyond the territory of the treated artery per participant	1 (0,2)

*Note:* Unless otherwise indicated, data are the numbers of participants, with percentages in parentheses.

^a^ For 30 participants with new ischemic brain lesions after carotid artery stenting treatment.

^b^ For the number of new ischemic brain lesions after carotid artery stenting Treatment. Data in parentheses are percentages.

We then analyzed the distribution of new ischemic brain lesions between the high and low PET uptake groups, finding no significant differences (in the territory of the treated artery only: 30.8% vs. 41.2%; beyond the territory of the treated artery only: 15.4% vs. 0%; in the mixed area: 53.8% vs. 58.8%; *p* = 0.318). Other detailed distribution counts are presented in Table S3.

### 3.3 High PET Uptake/ High SCAIL Score and New Ischemic Lesions Within 3 Days

**FIGURE 2:**
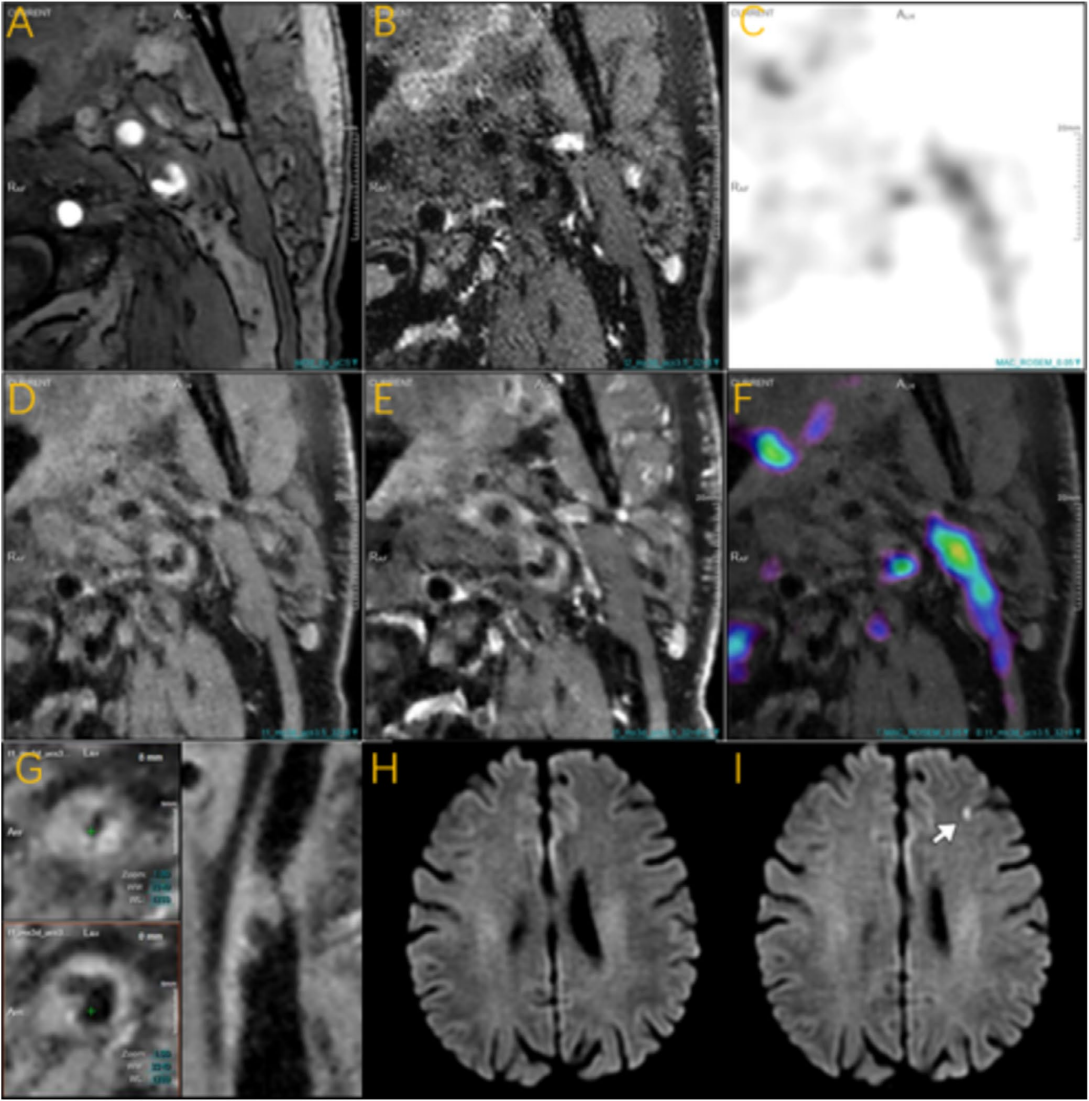
A male ages 60–70 years old with severe carotid stenosis treated by CAS. Images from A to sequentially demonstrate the carotid atherosclerotic plaque in this patient using different PET/MR sequences ((A) Time‐of‐flight MR angiography (TOF‐MRA); (B) T2‐weighted imaging (T2WI); (C) PET metabolic map; (D) T1‐weighted imaging (T1WI); (E) T1‐weighted contrast‐enhanced imaging (T1+C); (F) Fusion of T1WI and PET metabolic map; (G) Curved planar reformation (CPR) reconstruction; (H) Pre‐treatment brain diffusion‐weighted imaging (DWI); (I) Post‐treatment brain diffusion‐weighted imaging (DWI)), The surface of the plaque is irregular, showing a lipid‐rich necrotic core and ulceration, consistent with a Type VI—complex plaque in the AHA‐LT classification. The SUVmax is 3.16 and the TBR is 3.67, indicative of severe uptake. DWI of the brain before CAS (H) shows normal findings, DWI of the brain after CAS (I) at the same level shows new ipsilateral ischemic lesion in the left frontal lobe (white arrow).

Previous comparisons between groups showed that the proportion of high PET uptake and high SCAIL scores was significantly higher in the new ischemic lesions group compared to the no new ischemic lesions group. Representative cases are shown in Figure 2. We evaluated each of these indicators separately, in multiple binary logistic regression analysis, adjusting for demographics and cardiovascular risk factors (sex, age, hypertension, diabetes, hypercholesterolemia) in model 2, high PET uptake was independently associated with new ischemic lesions (aOR = 6.92, 95% CI: 1.35, 57.62; *p* = 0.036). Further adjustment for imaging variables of vascular lesion severity (plaque burden, positive remodeling, AHA‐VI plaque) in model 3 confirmed this association (aOR = 7.26, 95% CI: 1.22, 73.59; *p* = 0.049). Similarly, high SCAIL scores were independently associated with new ischemic lesions after adjustments in both models (model 2: aOR = 5.94, 95% CI: 1.51, 28.85; *p* = 0.016; model 3: aOR = 7.06, 95% CI: 1.50, 44.18; *p* = 0.020) (Table 3).

**TABLE 3:** Multiple binary logistic regression analysis.

Characteristic	Model 1			Model 2			Model 3		
	OR	95% CI	*p*	OR	95% CI	*p*	OR	95% CI	*p*
High PET uptake	5.74	1.30, 40.62	0.037^a^	6.92	1.35, 57.62	0.036^a^	7.26	1.22, 73.59	0.049^a^
High SCAIL score	5.61	1.56, 24.06	0.012^a^	5.94	1.51, 28.85	0.016^a^	7.06	1.50, 44.18	0.020^a^

*Note:* Model 1: no covariates were adjusted. Model 2: adjusted for Gender, Age, Hypertension, Diabetes and Hypercholesterolemia. Model 3: model 2+ plaque burden, Positive remodeling, and AHA‐VI plaque.

Abbreviations: CI, confidence interval; OR, odds ratio; SCAIL, symptomatic carotid atheroma inflammation lumen‐stenosis.

^a^ Statistically significant (*p* < 0.05).

**FIGURE 3:**
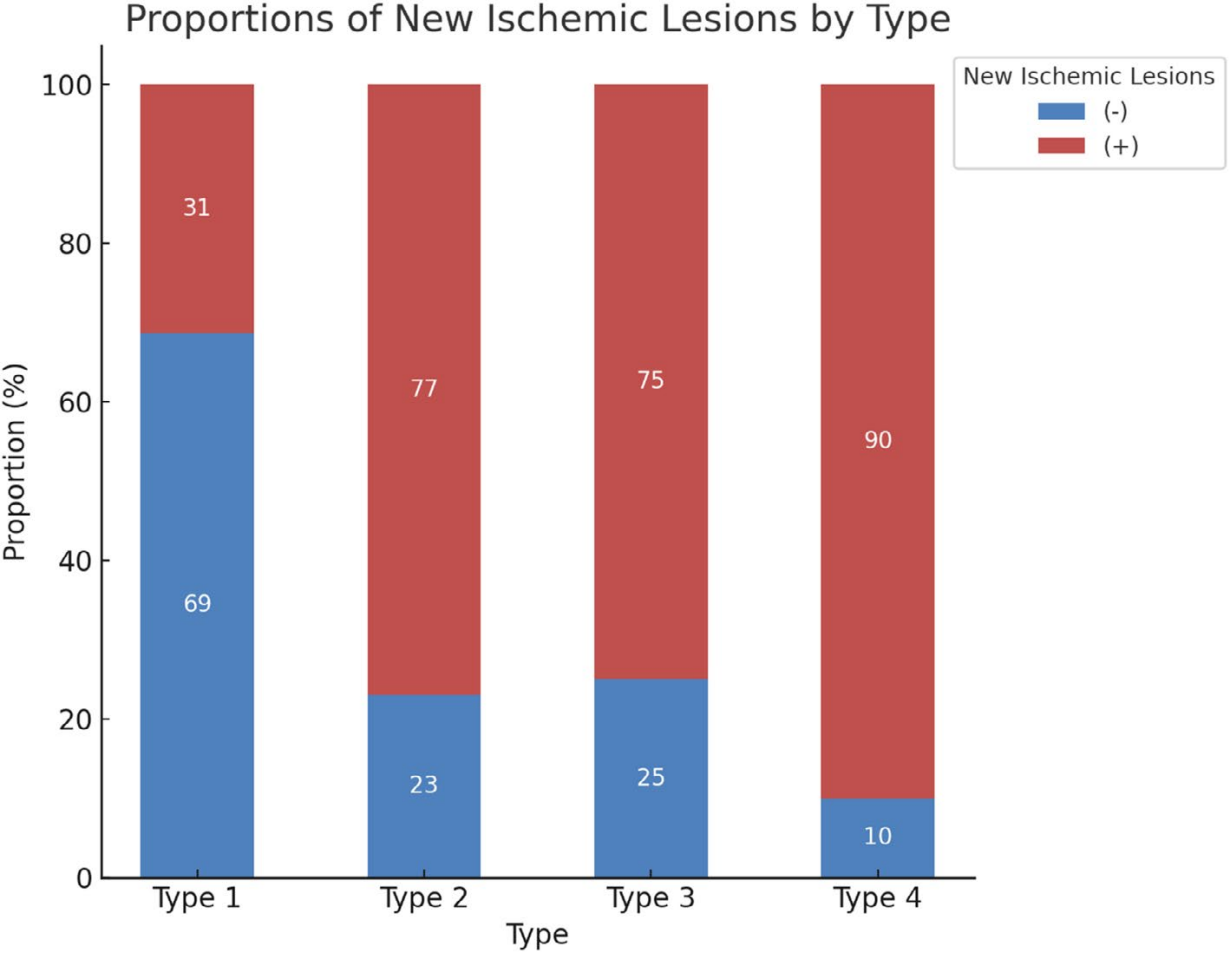
Distribution of different types in relation to new ischemic lesions. New ischemic lesions most frequently occurred in the type 4 group (*p* < 0.001).

Given the close relationship between PET uptake and SCAIL scores with complex plaques [18], we created a new variable “type” by multiplying the two categorical variables: Type 1 = high SCAIL score(−) + AHA‐VI plaque(−), Type 2 = high SCAIL score(+) + AHA‐VI plaque(−), Type 3 = high SCAIL score(−) + AHA‐VI plaque(+), and Type 4 = high SCAIL score(+) + AHA‐VI plaque(+). Additional multivariable analysis showed that Type 4 was a strong predictor of new ischemic lesions after similar adjustments (aOR = 25.52, 95% CI: 2.79, 655.25; *p* = 0.013) (Table 4). A bar chart illustrated that the proportion of new ischemic lesions was highest in the Type 4 group (90% vs. 10%) and lowest in the Type 1 group (31% vs. 69%) (Figure 3).

**TABLE 4:** Multiple binary logistic regression analysis by different types.

Characteristic type	Model 1			Model 2			Model 3		
	OR	95% CI	*p*	OR	95% CI	*p*	OR	95% CI	*p*
1	—	—		—	—		—	—	
2	7.33	1.51, 45.33	0.019^a^	9.56	1.66, 78.49	0.019^a^	9.96	1.71, 84.86	0.018^a^
3	6.60	1.09, 57.60	0.054	16.84	1.82, 286.80	0.025^a^	9.59	1.16, 129.35	0.053
4	19.80	2.68, 420.51	0.012^a^	27.92	3.03, 737.83	0.011^a^	25.52	2.79, 655.25	0.013^a^

*Note:* Type1 = high SCAIL score(−) + AHA‐VI plaque(−), Type2 = high SCAIL score(+) + AHA‐VI plaque(−), Type3 = high SCAIL score(−) + AHA‐VI plaque(+), Type1 = high SCAIL score(+) + AHA‐VI plaque(+). Model 1: no covariates were adjusted. Model 2: adjusted for Gender, Age, Hypertension, Diabetes, and Hypercholesterolemia. Model 3: model 2+ plaque burden, Positive remodeling.

Abbreviations: CI, confidence interval; OR, odds ratio; SCAIL, symptomatic carotid atheroma inflammation lumen‐stenosis.

^a^ Statistically significant (*p* < 0.05).

## 4 Discussion

Our study demonstrated three main findings. Firstly, a significantly higher proportion of AHA type VI plaques, high PET uptake, and high SCAIL scores were observed in participants who developed new ischemic brain lesions post‐CAS. Secondly, we analyzed the distribution patterns of these new ischemic lesions, noting that they were more likely to occur both within and beyond the treated artery territory and in both deep and peripheral brain areas. Thirdly, we found that high PET uptake and high SCAIL scores were independent predictors of new ischemic lesions during the perioperative period, with the predictive power enhanced when combined with plaque component analysis. Numerous studies [13, 32] have highlighted the significance of PET or complex plaques in detecting acute neurovascular syndrome and predicting recurrent strokes. To our knowledge, this is one of the few studies utilizing integrated PET/MR to assess patients undergoing CAS and predict perioperative ischemic lesions.

Numerous studies in recent years have highlighted the significance of imaging biomarkers in predicting ischemic events following carotid revascularization. Recent studies demonstrated that new ischemic brain lesions detected on MRI after carotid revascularization are associated with an increased long‐term risk of stroke, underscoring the prognostic value of imaging‐detected lesions in identifying high‐risk individuals [3]. Similarly, Kelly et al. developed a risk score that integrates carotid plaque inflammation measured by 18F‐FDG PET with stenosis severity, effectively identifying patients at higher risk for recurrent stroke after CAS. These studies underscore the importance of using advanced imaging biomarkers for risk stratification in patients undergoing CAS [23]. Our study highlights the significant role of PET/MR imaging in evaluating early ischemic brain injury post‐carotid artery stenting (CAS). PET is crucial for assessing inflammation, and integrating PET with high‐resolution vessel wall imaging in PET/MR enables simultaneous evaluation of metabolic activity and plaque composition. This approach offers higher precision and classification capability compared to PET/CT, enhancing component identification and overcoming the limitations of high‐resolution vessel wall imaging alone, which cannot accurately capture inflammatory metabolic information. In our study, 65% of participants developed new ischemic lesions following CAS, with the majority being asymptomatic. This finding aligns with the studies by a substudy of the International Carotid Stenting Study [5, 33], which reported similar rates of new lesion development post‐stenting. In contrast, our study enhances accuracy and precision through the use of PET/MR imaging.

In our comparison between groups, we found that clinical risk factors, symptomatology, and vessel wall and lumen‐related parameters (such as stenosis severity, plaque burden, and positive remodeling) were not associated with new ischemic lesions. For example, the stenosis severity was 63.3% in the new lesion group and 58.8% in the no new lesion group, with no statistically significant difference (*p* > 0.05). However, some studies on plaque burden have shown significant correlations with long‐term functional outcomes and recurrent strokes [34], suggesting that biomarkers of short‐term and long‐term prognosis may differ. The remodeling pattern of the vessel has also received widespread attention in recent years, providing important insights for vascular treatment. Positive remodeling is often associated with the formation of vulnerable plaques and acute ischemic stroke [35, 36]. Recent studies on the basilar artery have found a strong correlation between the remodeling index and plaque burden [36]. However, the remodeling pattern may not impact the outcomes of ischemic events and new ischemic cerebral lesions, offering new perspectives on evaluating vascular pathology [37].

However, the classification of plaque components, particularly the identification of complex plaques, showed significant differences between groups, highlighting the importance of plaque composition in risk stratification. The proportion of complex plaques was significantly higher in the new lesion group (50.0% vs. 17.6%, *p* = 0.028), consistent with the recent research [13], which found an association between complex plaques and a high risk of recurrent ischemic stroke or TIA. A study by Kwee [38] demonstrated that the presence of a lipid‐rich necrotic core in patients with carotid atherosclerosis and stroke was associated with recurrent cerebrovascular ischemic events. In a prospective multicenter study [39], intraplaque hemorrhage and total plaque volume were identified as independent risk factors for ipsilateral ischemic event recurrence in patients with mild to moderate carotid stenosis. These findings suggest that the impact of plaque composition on stroke recurrence and ischemic brain injury warrants further investigation. High PET uptake was also identified as a significant independent risk factor [40], indicating that CAS treatment carries a considerable risk of new ischemic lesions in the context of high inflammatory expression, necessitating further personalized therapeutic guidance. The SCAIL score, which combines metabolic activity with stenosis severity, has been applied to predict future stroke recurrence. Traditionally, treatment decisions were based solely on stenosis severity, but this approach posed substantial recurrence risks, especially for patients with moderate stenosis. In such cases, decisions regarding revascularization are often nuanced, requiring new methods to identify the most suitable candidates. Previous studies [23, 26] have shown that the risk of stroke recurrence increases with higher SCAIL scores, consistent with our findings. When combining metabolic and plaque component analyses, the predictive capability for new ischemic lesions improves further. Although our sample size was small, our results underscore the potential of using advanced imaging biomarkers to identify patients at higher risk for adverse outcomes post‐CAS. This approach could help clinicians better assess post‐stenting risks and optimize treatment strategies. Large‐scale clinical studies are needed to confirm the stratification capabilities and clinical utility of these combined biomarkers in predicting stroke recurrence and ischemic brain injury.

Regarding the distribution patterns of new ischemic lesions, our study found similarities with previous research. Kim et al. [41] reported that in patients with ICAS, 51% of new lesions post‐stenting were located solely within the stented vessel. Our study also found that some new ischemic brain injuries were confined to the treated artery territory (25.5%), while others were beyond the treated artery (2%), and a significant portion was in mixed territories (36.2%). This distribution suggests that endovascular treatment may result in widespread ischemic damage throughout the brain, even in areas not directly manipulated during the procedure. This indicates that emboli might be dispersed via the Willis circle or other mechanisms, leading to a widespread distribution of ischemic brain injuries. Our study also indicates that post‐CAS treatment, most new lesions may simultaneously affect both the peripheral cortical and subcortical regions and the deep white matter areas. This supports embolization as the primary mechanism of new lesion formation [41], consistent with findings by the International Carotid Stenting Study [4]. However, the injury mechanisms might differ slightly from those observed with intracranial stenting procedures.

Our results show that patients with high PET uptake and complex plaque characteristics had a higher incidence and broader range of new ischemic lesions. This suggests that inflammation and plaque instability are key factors in post‐procedural embolic events. These findings align with previous studies [42, 43] which demonstrated that 18F‐FDG uptake in plaques is associated with macrophage infiltration and metabolic activity, both of which destabilize atherosclerotic plaques and increase their susceptibility to rupture or micro‐embolization. Complex plaques, such as AHA type VI lesions, often contain intraplaque hemorrhage, a lipid‐rich necrotic core, and a disrupted fibrous cap, features that facilitate the release of small emboli into the cerebral circulation. These emboli can occlude small downstream vessels, manifesting as new ischemic lesions on DWI, even in the absence of overt clinical symptoms. Thus, the interplay between plaque composition, inflammation, and embolic propensity provides a plausible mechanistic framework linking the imaging findings to the observed perioperative ischemic complications. This underscores the importance of preoperative plaque inflammation assessment for more precise patient stratification and treatment decisions. In treating patients with carotid artery stenosis, a more comprehensive evaluation is necessary, rather than solely relying on symptoms and stenosis severity. Understanding the inflammatory activity within plaques preoperatively can significantly impact patient stratification and therapeutic strategies.

While our study benefits from a comprehensive evaluation of clinical and imaging biomarkers, it also has several limitations. First, the relatively small sample size limits the generalizability of our findings. Second, the retrospective design may introduce selection bias, although we sought to minimize this through stringent inclusion criteria. Third, the low number of symptomatic ischemic events (only 2 cases) restricts our ability to draw definitive conclusions regarding the prediction of symptomatic outcomes based on imaging biomarkers, which highlight the need for larger, prospective studies to better understand the relationship between plaque characteristics and symptomatic ischemic events. Additionally, despite the high inter‐observer and intra‐observer consistency in radiological measurements, the possibility of measurement variability cannot be entirely excluded. Lastly, our study focused primarily on the perioperative period and did not assess long‐term outcomes, which warrant further investigation.

In conclusion, our study highlights the significant predictive value of PET/MR‐related indicators in identifying the risk of new ischemic lesions during the perioperative period following carotid artery stenting. High PET uptake and SCAIL scores, particularly when combined with plaque component analysis, serve as robust predictors of ischemic risk. These findings underscore the potential of integrated PET/MR imaging in improving risk stratification and guiding clinical management in patients undergoing CAS. Further large‐scale, prospective studies are needed to validate these findings and confirm the clinical utility of these imaging biomarkers.

## Conflicts of Interest

The authors declare no Conflicts of Interest.

## Supporting information


**Figure S1.** Patient selection flowchart.

**Table S1.** AHA‐LT Classification Used for MR plaque imaging.
**Table S2.** SCAIL item measures and points.
**Table S3.** Comparison of New ischemic brain lesions at diffusion‐weighted MRI between High PET uptake or not.

## Data Availability

Research data are not shared.
