# Supplementary figures and images for: Association of Plaque Characteristics With New Ischemic Lesions After Carotid Artery Stenting
Source: CNS Neurosci Ther. 2025 Mar 3;31(3):e70312. doi: 10.1111/cns.70312 (PMC11875062; doi:10.1111/cns.70312)

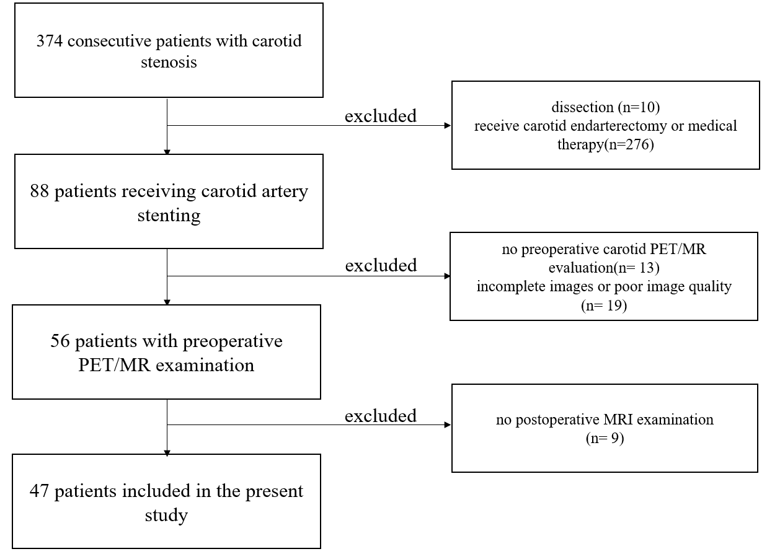

Supplement: Supplementary file 1 — Figure S1. Patient selection flowchart. [file CNS-31-e70312-s001.tif]
