# Supplementary material for: Association of Plaque Characteristics With New Ischemic Lesions After Carotid Artery Stenting
Source: CNS Neurosci Ther. 2025 Mar 3;31(3):e70312. doi: 10.1111/cns.70312 (PMC11875062; doi:10.1111/cns.70312)
Supplement: Supplementary file 2 — Table S1. AHA‐LT Classification Used for MR plaque imaging. Table S2. SCAIL item measures and points. Table S3. Comparison of New ischemic brain lesions at diffusion‐weighted MRI between High PET uptake or not. [file CNS-31-e70312-s002.docx]

Table S1: AHA-LT Classification Used for MR Plaque Imaging

| AHA-LT | Definition |
| --- | --- |
| AHA-LT I/II | near-normal wall thickness |
| AHA-LT III | diffuse intimal thickening or small eccentric plaque, no calcification |
| AHA-LT IV/V | plaque with a lipid or necrotic core surrounded by fibrous tissue with possible calcification |
| AHA-LT VI | complex plaque with possible surface defect, hemorrhage, or thrombus |
| AHA-LT VII | calcified plaque |
| AHA-LT VIII | fibrotic plaque without lipid core and with possible small calcifications |

Magnetic resonance (MR) imaging allows for differentiation of the following American Heart Association lesion types (AHA-LT) in carotid arteries: AHA-LT I/II, AHA-LT III, AHA-LT IV/V, AHA-LT VI, AHA-LT VII, and AHA-LT VIII.

Table S2: SCAIL Item Measures and Points

| SCAIL item | Measure | SCAIL points |
| --- | --- | --- |
| Plaque SUVmax, g/mL | <2 | 0 |
|  | 2–2.99 | 1 |
|  | 3–3.99 | 2 |
|  | ≥4 | 3 |
| Lumen stenosis, % | <50 | 0 |
|  | 50–69 | 1 |
|  | ≥70 | 2 |
| Total |  | 0–5 |

Abbreviations: SCAIL = symptomatic carotid atheroma inflammation lumen-stenosis; SUVmax = maximum standardized uptake value.

Table S3: Comparison of New Ischemic Brain Lesions at Diffusion-weighted MRI between High PET uptake or not

| **Characteristic** | **High PET uptake** | | **p-value** |
| --- | --- | --- | --- |
|  | **no, N = 32^1^** | **yes, N = 15^1^** |  |
| **New Ischemic Lesions** | 17 (53.1%) | 13 (86.7%) | 0.026 |
| **Symptomatic** | 1 (3.1%) | 1 (6.7%) | 0.541 |
| **Distribution of new lesions** |  |  | 0.318 |
| In the territory of the treated artery only | 7 (41.2%) | 4 (30.8%) |  |
| Beyond the territory of the treated artery only | 0 (0.0%) | 2 (15.4%) |  |
| in the mixed area | 10 (58.8%) | 7 (53.8%) |  |
| **Location of new lesions*** |  |  | 0.440 |
| Peripheral brain areas only | 5 (29.4%) | 4 (30.8%) |  |
| Deep brain areas only | 2 (11.8%) | 4 (30.8%) |  |
| Peripheral and deep brain areas | 10 (58.8%) | 5 (38.5%) |  |
| **No. of new lesions per participant**^#^ | 1 (0, 3) | 2 (1, 3.5) | 0.167 |
| No. of new lesions in the territory of the treated artery per participant | 1 (0, 2) | 1 (0.5, 2) | 0.445 |
| No. of new lesions beyond the territory of the treated artery per participant | 0 (0, 1) | 1 (0, 2) | 0.088 |
| Note.—Unless otherwise indicated, data are numbers of participants, with percentages in parentheses.  * For 30 participants with new ischemic brain lesions after carotid artery stenting treatment.  ^#^ For the number of new ischemic brain lesions after endovascular treatment. Data in  parentheses are percentages. | | | |
